# Intervention and Evaluation of Mobile Health Technologies in Management of Patients Undergoing Chronic Dialysis: Scoping Review

**DOI:** 10.2196/15549

**Published:** 2020-04-03

**Authors:** Yang Yang, Helen Chen, Hammad Qazi, Plinio P Morita

**Affiliations:** 1 School of Public Health and Health Systems Faculty of Applied Health Sciences University of Waterloo Waterloo, ON Canada

**Keywords:** mobile health, renal dialysis, health technology assessment, patient outcome assessment

## Abstract

**Background:**

Studies have shown the effectiveness and user acceptance of mobile health (mHealth) technologies in managing patients with chronic kidney disease (CKD). However, incorporating mHealth technology into the standard care of patients with CKD still faces many challenges. To our knowledge, there are no reviews on mHealth interventions and their assessments concerning the management of patients undergoing dialysis.

**Objective:**

This study provided a scoping review on existing apps and interventions of mHealth technologies in adult patients undergoing chronic dialysis and identified the gaps in patient outcome assessment of mHealth technologies in the literature.

**Methods:**

We systematically searched PubMed (MEDLINE), Scopus, and the Cumulative Index to Nursing and Allied Health Literature databases, as well as gray literature sources. Two keywords, “mHealth” and “dialysis,” were combined to address the main concepts of the objectives. Inclusion criteria were as follows: (1) mHealth interventions, which are on a smartphone, tablet, or web-based portals that are accessible through mobile devices; and (2) adult patients (age ≥18 years) on chronic dialysis. Only English papers published from January 2008 to October 2018 were included. Studies with mHealth apps for other chronic conditions, based on e-consultation or videoconferencing, non-English publications, and review papers were excluded.

**Results:**

Of the 1054 papers identified, 22 met the inclusion and exclusion criteria. Most studies (n=20) were randomized controlled trials and cohort studies. These studies were carried out in 7 countries. The main purposes of these mHealth interventions were as follows: nutrition or dietary self-monitoring (n=7), remote biometric monitoring (n=7), web-based portal (n=4), self-monitoring of in-session dialysis-specific information (n=3), and self-monitoring of lifestyle or behavioral change (n=1). The outcomes of the 22 included studies were organized into five categories: (1) patient satisfaction and acceptance, (2) clinical effectiveness, (3) economic assessment, (4) health-related quality of life, and (5) impact on lifestyle or behavioral change. The mHealth interventions showed neutral to positive results in chronic dialysis patient management, reporting no to significant improvement of dialysis-specific measurements and some components of the overall quality of life assessment. Evaluation of these mHealth interventions consistently demonstrated evidence in patients’ satisfaction, high level of user acceptance, and reduced use of health resources and cost savings to health care services. However, there is a lack of studies evaluating safety, organizational, sociocultural, ethical, and legal aspects of mHealth technologies. Furthermore, a comprehensive cost-effectiveness and cost-benefit analysis of adopting mHealth technologies was not found in the literature.

**Conclusions:**

The gaps identified in this study will inform the creation of health policies and organizational support for mHealth implementation in patients undergoing dialysis. The findings of this review will inform the development of a comprehensive service model that utilizes mHealth technologies for home monitoring and self-management of patients undergoing chronic dialysis.

## Introduction

### Background

Mobile technologies have changed the way individuals communicate and are also transforming the health ecosystem by providing patients and health care providers a wide range of supportive tools for monitoring and managing health information, thereby facilitating better delivery of health care services [1]. As a subdomain in the digital health field, mobile health (mHealth) is defined as “medical and public health practice through the use of mobile devices, such as mobile phones, patient monitoring devices, personal digital assistants (PDAs) and other wireless devices” [2]. More specifically, the mobile technology refers to the “wireless devices and sensors (including mobile phones) that are intended to be worn, carried, or accessed by the person during normal daily activities” [3]. This definition was adopted in this study.

According to the World Health Organization, mHealth technologies have four major application scenarios, including chronic disease monitoring, health management, web-based diagnosis and treatment, and medical appointment scheduling [2]. mHealth technologies have been used to monitor and manage chronic conditions, such as in heart disease, diabetes, hypertension, stroke, asthma, dementia, chronic pain, chronic pulmonary obstructive disease, and chronic kidney disease [4-7]. Dialysis is a medical intervention for patients with end-stage chronic kidney disease. These patients have already suffered a loss of independence by spending a significant amount of time on dialysis treatment in-center or at home [8,9]. No other chronic health condition has such enormous physical and cognitive effects on patients’ daily lives [9]. Solutions aiming at reducing time on visiting dialysis centers are especially beneficial to this population, which reflects patient-centered care principle as well as improves patients’ quality of life [10]. mHealth technologies can expedite patient communication and facilitate home monitoring and self-management, with the potential of improving patient’s overall well-being. However, there is no systematic review on existing literature on mHealth solutions and their effectiveness and benefits in adult chronic dialysis patient management.

The Health Technology Assessment Core Model (HTA-CM), a general framework that facilitates international collaboration of HTA, divides the assessment outcomes into 9 domains, including “health problem and current use of the technology; description and technical characteristics of technology; safety; clinical effectiveness; costs, economic evaluation; ethical analysis; organizational aspects; social aspects; and legal aspects” [11]. The 9-domain HTA-CM has been used in this study, with adult patients undergoing chronic dialysis as a use case for mHealth technologies. Siddique et al [12] evaluated apps of managing CKD using the Mobile App Rating Scale. However, the evaluation of these apps was done by a team of reviewers, mainly on the construction of apps instead of the efficacy and benefits of using apps in patient care and clinical settings.

### Objectives

This study aimed to provide a comprehensive review of existing mHealth interventions for adult patients undergoing chronic dialysis and to identify the gaps in the outcome evaluation of mHealth technologies in the literature. The objectives of this study are to (1) summarize the categories of interventions and their main functions of mHealth technologies in the management of adult patients undergoing chronic dialysis through reviewing the existing literature in a systematic approach, (2) examine how these mHealth interventions are evaluated, and (3) identify gaps in the assessment of mHealth technologies in this population.

## Methods

### Study Design

This study used the five-stage methodological framework for scoping reviews as outlined by Arksey and O’Malley [13], involving (1) identifying the research question, (2) identifying relevant studies, (3) study selection, (4) charting the data, and (5) collating, summarizing, and reporting the results. The 27-item Preferred Reporting Items for Systematic Reviews and Meta-Analysis (PRISMA) extension for scoping reviews was employed as the protocol for this study [14].

### Identifying the Research Questions

The study population was adult patients undergoing chronic dialysis, and intervention types were services utilizing mHealth technology. Research questions were developed based on an initial literature search and were refined iteratively during the discussions among the research team. The research questions were as follows: (1) what mHealth apps exist to support adult chronic dialysis patients, and (2) how are these mHealth solutions evaluated?

### Identifying Relevant Studies

A systematic search strategy was employed to identify relevant literature to the research questions. Two keywords, “mHealth” and “dialysis,” were combined to address the two main concepts of the research questions (see Multimedia Appendix 1). The search was performed on PubMed (MEDLINE), Scopus, and the Cumulative Index to Nursing and Allied Health Literature (CINAHL) databases. Furthermore, gray literature was explored from the Canadian Agency for Drugs and Technologies in Health (CADTH)’s “Grey Matters,” Health Quality Ontario, Food and Drug Administration, Ottawa Hospital Research Institute, Pan Canada HTA Collaborative, International Information Network on New and Emerging Health Technologies, and Google Scholar and Google search (see Multimedia Appendix 2). In the CADTH’s “Grey Matters,” the HTA section was used for all countries to find relevant papers. The first 40 records of Google Scholar and Google search results, as well as those from searching the FDA database, were screened for relevant papers. Two independent researchers (YY and MZ) searched databases and gray literature for references of identified papers published from January 2008 up to October 15, 2018.

### Selecting Relevant Papers for the Review

The inclusion and exclusion criteria for this study are listed in Textbox 1. Papers retrieved from each database were imported into the RefWorks software, a web-based reference management software produced by Ex Libris (Jerusalem, Israel), a ProQuest company. Duplicates were removed, and the title and abstract of each paper were screened by YY and JS. They assessed each paper as *included*, *excluded*, or *unsure*. Where there is uncertainty in achieving an agreement (ie, those marked as “unsure” or classified into different categories by 2 reviewers), a full-text review was conducted to determine whether they should be included. The individual screening results were compared, and discrepancies were resolved by consensus through discussion among the research team members. The full-text review was conducted on papers that met the inclusion criteria from the title and abstract review.

**Inclusion and exclusion criteria.**

Inclusion criteria were as follows:

- Adult patients both males and females (age ≥ 18 years)
- Receiving chronic dialysis (in-center or at home)
- Using mobile health (mHealth) interventions
- Papers found using search strategy started from January 2008 to October 2018 appearing at this time frame
- English papers only

Exclusion criteria were as follows:

- Participants aged <18 years (pediatric, adolescent)
- mHealth interventions based on e-consultation or videoconferencing
- Study protocols have no preliminary results
- Non-English publications
- Review papers

### Charting the Data

The research team collaboratively developed the data charting form and determined the variables for extraction. The descriptive charting information includes (1) paper general description: first author and year, study design, study location, and patient population; and (2) intervention-specific information: intervention and mHealth app purpose, main functions, delivery method, duration and follow-up period, data collected, outcomes measured, and findings. Data extracted during full-text review of all selected papers are summarized in Multimedia Appendix 3.

### Collating, Summarizing, and Reporting the Results

The general description of the reviewed papers was collated according to the descriptive characteristics of studies. The thematic content analysis of interventions and associated outcomes for each study was performed by the research team after a concurrent review of the charted data. First, codes were developed and applied to analyze the data. The coded segments of all the charted data were then created with color-coded quotations. Furthermore, the code summary was organized into an Excel table for thematic content analysis. This table was sorted by codes and density, looking for recurrent patterns that were addressed by the included papers, both across the whole dataset to compare the included studies and within each study until the key themes were identified. The research findings were summarized with the outlined objectives of this study.

## Results

### Selection of Included Papers

**Figure 1:**
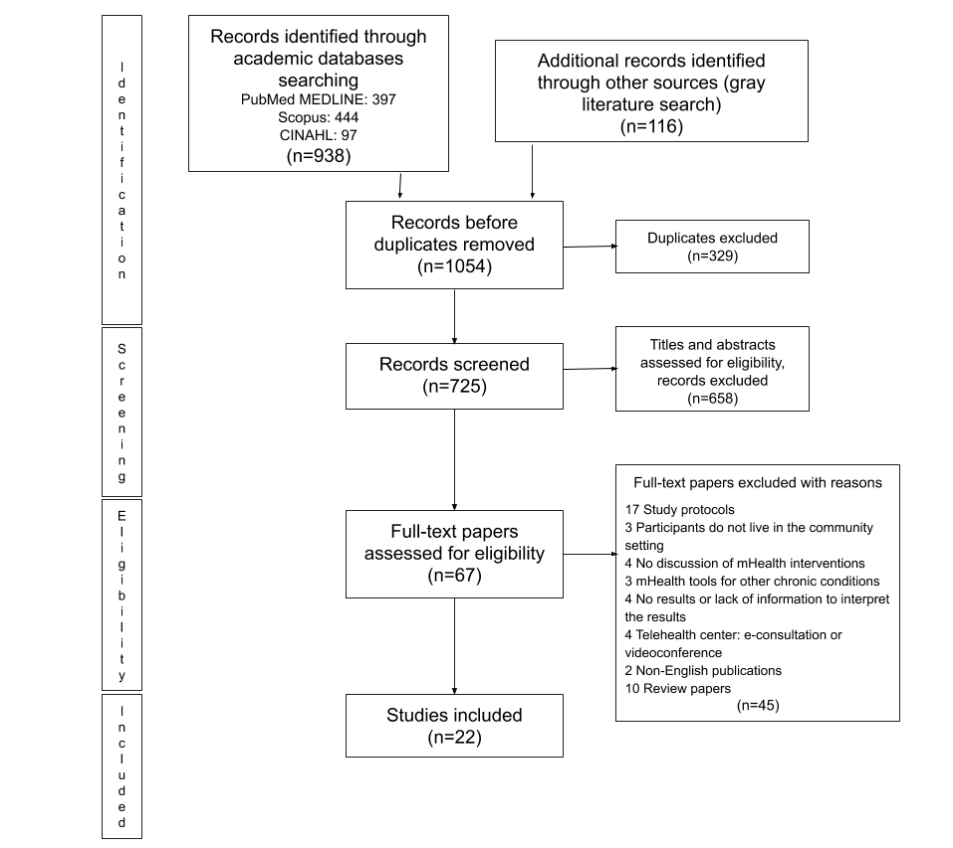
The Preferred Reporting Items for Systematic Reviews and Meta-Analysis flow diagram. mHealth: mobile health.

A total of 938 published papers were retrieved from the PubMed (MEDLINE), Scopus, and CINAHL databases, including one paper that was identified through a reference review of identified papers. The gray literature search resulted in 116 additional papers. Of the 1054 identified papers, 329 duplicates were excluded. Of the remaining 725 papers, 67 were selected for a full-text review based on the screening results of titles and abstracts. After the full-text review, 22 papers were included in this scoping review. Figure 1 shows the PRISMA flow diagram.

### General Characteristics of the Included Studies

Among 22 reported studies, cohort study was the most common study type (12 prospective and 4 retrospective studies), followed by randomized controlled trials (RCTs, 4 studies), 1 mixed method study, and 1 case study. The general characteristics of these studies are presented in Table 1.

**Table 1:** General characteristics of the included studies.

Characteristics			Studies (n=22)
**Study design**			
	Randomized controlled trial	4	
	Cohort (prospective)	12	
	Cohort (retrospective)	4	
	Others	2	
**Origin of study**			
	United States	13	
	Canada	2	
	Australia	2	
	India	2	
	Others	3	
**Year of publication**			
	2014-2018	13	
	2008-2013	9	

### Types of Current Mobile Health Interventions

Dialysis patient management services enabled by mHealth technology fall into five main categories: (1) nutrition or dietary self-monitoring (n=7; [15-21]), (2) remote biometric monitoring (RBM) (n=7; [22-28]), (3) web-based portal (n=4; [29-32]), (4) self-monitoring of in-session dialysis-specific information (n=3; [33-35]), and (5) self-monitoring of lifestyle or behavioral change (n=1; [36]). These mHealth interventions were delivered using various technology platforms, including smartphone (7 studies), home-monitoring or telemonitoring unit or telemetry system (6 studies), tablet (5 studies), PDA (4 studies), and fitness tracker (1 study). The mHealth interventions were applied to various dialysis modalities, including hemodialysis (HD, 10 studies), peritoneal dialysis (9 studies) and home hemodialysis (HHD, 4 studies). The study duration ranged from 2 weeks to 42 months.

### Outcome Evaluation of Current Mobile Health Interventions

The data collected by these studies included patient characteristics, dialysis in-session indicators, physiological measurements, quality-of-life indicators, health care resource utilization, change of lifestyle or behavior, and usage and user perceptions on mHealth interventions. The thematic content analysis identified five main themes in the evaluation of mHealth interventions: (1) patient satisfaction and acceptance, (2) clinical effectiveness, (3) economic assessment, (4) health related quality of life (HRQoL), and (5) impact on lifestyle or behavioral change. Table 2 lists outcome measures of current mHealth interventions.

**Table 2:** Outcome evaluation of current mobile health interventions.

Outcome measures (themes)	Studies (n=22)	Main functions (type of use)	Sample sizes (range)	Study duration (range)
Patient satisfaction and acceptance	14	Nutrition or dietary self-monitoring (6); self-monitoring of in-session dialysis-specific information (3); web-based portal (2); RBM^a^ (2); self-monitoring of lifestyle or behavioral change (1)	(9, 241)	2 weeks to >15 months
Clinical effectiveness	9	Nutrition or dietary self-monitoring (4); RBM (3); self-monitoring of in-session dialysis-specific information (1); web-based portal (1)	(1, 2424)	30 days to 2 years
Economic assessment	8	RBM (4); web-based portal (3); self-monitoring of in-session dialysis-specific information (1)	(22, 269)	6 to 42 months
HRQoL^b^	5	RBM (2); web-based portal (2); self-monitoring of in-session dialysis-specific information (1)	(20, 60)	2 weeks to 17 months
Impact on lifestyle or behavioral change	5	Nutrition or dietary self-monitoring (4); self-monitoring of lifestyle or behavioral change (1)	(1, 72)	30 days to 6 months

^a^RBM: remote biometric monitoring.

^b^HRQoL: heart-related quality of life.

The studies revealed that mHealth technology has a beneficial effect on dialysis-specific measures. mHealth solutions helped to improve behaviors and reduce the utilization of health resources. Patients in these studies expressed satisfaction and acceptance toward mHealth technologies in dialysis care.

#### Patient Satisfaction and Acceptance

Patients’ attitude toward mHealth interventions was assessed by Likert evaluation questionnaires at the end of the study period. Questions on the questionnaires include ease of use, reliability and performance of the app, perceived usefulness, and overall impression. The evaluation demonstrated affirmative results, reporting positive responses concerning satisfaction and acceptability (8 studies), the ability to understand and use the app (5 studies), confidence in the treatment (1 study), as well as access and accessibility (1 study).

A consistent rating of satisfaction and high level of acceptability was found in studies for self-monitoring of nutrition or dietary, in-session dialysis-specific information, RBM, and online portals, with median satisfaction scores rated over 4 of 5 and similar acceptability scores (approaching 4 of 5) [16,17,22,25,31,33,34]. In addition to high adherence throughout the follow-up reported by Stark et al [19], some studies also revealed that many participants would continue using mHealth interventions beyond the study periods [21,22,35,36]. Patients’ willingness to continue using mHealth apps beyond the study periods indicated a high level of acceptability to the mHealth intervention in this cohort. One study reported a positive feedback from the nurses about satisfaction with the remote monitoring system for patients undergoing HHD, referring it a “time-saving tool” [34], and another study demonstrated improved interactions with patients [18].

Of the 5 studies that reported participants’ ability to use the app, all the study results demonstrated that the apps were easy to use. This includes interfaces and scanner input mechanism [15], helpful feedback and easy-to-understand instructions [16,17], easy-to-follow interface demonstration while browsing the data [33], and app and web portals that are easy to navigate [31,32].

The use of mHealth technologies, especially facilitating RBM, was found associated with increased confidence in health care activities and reduced negative attitudes toward dialysis care [25]. One study reported patients’ confidence in the treatment, with increases in confidence in the treatment and decreased perception of “being a burden to the family” or “having kidney disease takes too much time from the patient’s life” [25]. Furthermore, one study evaluated access and accessibility, and most of the participants felt that they had a positive impression on getting access to a renal specialist [31].

#### Clinical Effectiveness

Nine of the included studies measured dialysis-specific information, including interdialytic weight gain (IWG), blood pressure (BP) pre- and postdialysis, ultrafiltration rate, laboratory tests (hemoglobin, serum potassium, serum phosphorus, serum parathyroid hormone [PTH], serum albumin level), and technique failure rates at baseline and follow-up. The results were identified from these studies, demonstrating neutral to positive results in patients using mHealth interventions, compared with their peers receiving standard care. Most of the results (6/9) showed nonsignificant differences between the baseline and follow-up in the intervention groups, whereas a few studies (3/9) demonstrated significant improvement of dialysis-specific measurement results throughout the intervention periods.

Four studies that examined IWG had inconclusive results, showing nonsignificant differences between the baseline and follow-up period [33], a strong trend for relatively lower IWG (*P*=.06) in the intervention groups during follow-up [16], a significant mean reduction in weight gain between the groups at the end of the study [26], and a superimposed linear regression of the reduction of average daily IWG for 1 participant during the follow-up in a case study [20]. Except for the case study, the differences in their sample sizes (n=20, n=44, and n=120, respectively) and respective study locations in three different regions (Japan, United States, and Germany, respectively) may have resulted in these inconclusive findings.

Hand et al [18] reported a significant change in high serum PTH between baseline and follow-up, its relatively longer follow-up of 6 months as opposed to 3 months and 6 weeks in other studies, as well as its 3-arm trial designed to separate the effects of algorithm from those of additional care time may account for its statistical significance. Neumann et al [36] showed significant differences on unfiltered ultrafiltration rate, duration on dialysis, and BP between groups at the end of the study. The relatively larger sample size (n=120) as compared with other studies with less than 50 participants may contribute to its significant results. No other studies with laboratory tests identified significant differences in the proportions of patients in the target ranges for hemoglobin, serum potassium, serum phosphorus, or serum albumin level between the start and end of the studies.

In addition to patients’ physiological measurements, 2 studies reported significant results in adjusted hazard ratios of all-cause attrition and 5-year survival comparing intervention groups vs matched controls [23,30].

#### Economic Assessment

Eight of the included studies measured financial aspects, primarily from changes in use of hospital or health care services, including the number of hospitalizations or emergency room (ER) or clinic visits (4 studies), the number of days in the hospital and associated costs (4 studies), as well as nursing time and travel time or distance (3 studies). These studies consistently demonstrated the economic benefit of mHealth technology in cost and resource reduction to health care service providers.

Four studies that evaluated the impact on the utilization of hospital or health care resources reported significant cost savings per study day by reduced number of hospital visits or ER visits and fewer hospital days [27,28], reduced frequency of nurse-initiated telephone contacts required for medical alerts [27], reduced outpatient visit claim payment amounts [24], as well as lower medication cost [29]. The other 4 studies assessed the change in nursing time and telephone usage, as well as clinical interventions from generated alerts using mHealth apps. The results showed that remote reviews contributed to reduced telephone use [31] and savings of nursing and patient travel times and travel distance from the avoidance of conducting home and (or) unit visits [32,34]. These remote reviews also resulted in changes to dialysis prescriptions, mainly related to adjustments in patients' dry weight [32,34], avoidance of admissions, medication changes, referrals or advice from a dietician or pharmacy, and self-management at home [22].

#### Health-Related Quality of Life

Health-related quality of life (HRQoL) was measured using the Kidney Disease Quality of Life-36 (KDQOL-36; 2 studies), the 36-Item Short Form Health Survey (SF-36; 2 studies), and the EuroQol Five Dimensions Questionnaire (1 study), with inconclusive study results. Most of the studies (4/5) showed no significant improvement in quality of life (QoL) throughout the study periods. One study demonstrated a considerable improvement on the Social Functioning Score, but all the other KDQOL scores had no significant difference in the intervention group [33]. Another study revealed mixed results with improved SF-12 Physical Health Composite scores, but slightly decreased scores on symptom or problem list, effects of kidney disease, burden of kidney disease, and SF-12 Mental Health Composite at exit [22].

The mixed results on the effects of HRQoL may partly be attributed to the use of varying assessment instruments in different studies; for example, the 2 cohort studies reported QoL utilizing SF-36 and KDQOL-36 separately. SF-36 is recognized as a generic assessment tool that measures QoL for any type of disease, whereas KDQOL-36 is a disease-specific instrument assessing patients with kidney disease. The first 12 items of KDQOL-36 consist of core items that are equivalent to SF-12, and the remaining items assess the burden symptoms and effects of kidney disease [22].

#### Impact on Lifestyle or Behavioral Change

Five of the included studies reported patients’ lifestyle or behavioral change by measuring sodium intake (2 studies), calcium intake (2 studies), self-efficacy on diet proportion estimation (2 studies), or physical activity level and sleep (1 study). Of the 5 studies, 4 studies reported a decreasing trend of calcium and sodium intake and an increasing dietary self-efficacy. One study showed no significant difference in physical activity levels for patients undergoing HD using an off-the-shelf fitness tracker in the intervention group who received the feedback, compared with the control group with no feedback provided [36].

Welch et al [16] showed a significant decrease in sodium intake and calories, a marginal decrease in calories (*P*=.09) across all patients in the intervention group, and a marginal decrease in protein (*P*=.08) among active users who used mHealth app more than half of the time during the study.

Two studies that reported self-efficacy had slightly different results. One reported that most of the participants had an improvement in their self-efficacy in pre- and poststudy assessments [17], whereas another one showed no significant improvement between groups or over time in participants’ self-efficacy [16]. It revealed that improved behaviors in daily life might not be supported by self-efficacy. While examining perceived control, Welch et al [16] demonstrated a significant group difference with time, where a higher perceived control was reported in the intervention group than the control group at the end of self-monitoring period.

## Discussion

### Principal Findings

This scoping review summarized the literature on current mHealth technologies in chronic dialysis patient management, providing the types of intervention, outcome evaluation, and the gaps in outcome evaluation of mHealth technology that can be addressed in future research. The high satisfaction ratings are in line with other studies evaluating adherence of mHealth tools in renal transplant recipients [37,38], in elderly, low-income, and vulnerable patient population [8].

### Advantages of Mobile Health Technologies for Chronic Dialysis

From the patients’ perspectives, mHealth interventions demonstrated a great potential to facilitate the monitoring of symptoms, improve self-management–related physical or psychosocial consequences and associated comorbidities, maintain compliance with dialysis prescription, and improve lifestyle while receiving chronic dialysis [39]. From the health care providers’ perspective, rapid advancement in mobile technology enables real-time data capture and exchange between patient self-monitoring devices and a remote monitoring system. This creates opportunities to analyze the data and provide prompt feedback to patient-generated alerts [40].

Nayak et al [40] summarized the existing remote monitoring platforms used to support home dialysis modalities, identifying the system capacity being “two-way, rapid, real-time communication to help troubleshoot problems.” Similarly, Jeddi et al [41] demonstrated that the features and functionalities of computerized systems on self-management outcomes of patients with CKD include “inform, record, display, communicate, remind or alert and guide.” Digital IT solutions, particularly, mHealth interventions, have a unique advantage to meet this need.

The 22 included studies were multifaceted with respect to general characteristics and intervention-specific mHealth technologies. In a review paper, Havas et al [37] identified 10 aspects of self-management interventions from a patient’s perspective, suggesting a complex and multifactorial framework of self-management in patients with CKD. Our findings are in line with some domains, such as getting into routines and using reminder systems, monitoring weight, tracking fluid and food, and modifying lifestyle (ie, self-monitoring of physical activity and sleep).

In this review, 10 of the included studies utilized mHealth interventions for RBM and self-monitoring of in-session dialysis, which is an essential and integral part of self-management in patients undergoing dialysis. This provides a good indication of good interest and motivation of using mHealth technologies facilitating home monitoring and self-care. The web-based portals that have been identified from this review provide good evidence as mHealth interventions being utilized by both the patients undergoing dialysis as well as their clinical coordinators. Similarly, using commercially available fitness trackers can quantify physical activity levels and sleep data in patients undergoing dialysis, providing the opportunity of promoting a healthier lifestyle in this population.

Patients undergoing dialysis are often required to make signiﬁcant changes in their dietary intake. A review paper reveals that mHealth nutrition apps usually lead to better adoption of self-monitoring and changes in dietary intake compared with conventional techniques [42]. Due to the complication of dietary adjustments, the restriction on fluid and dietary intake is a major stressor for this cohort [43]. The mHealth interventions for self-management of dietary intake have become increasingly available, assisting in recording food and ﬂuid intake for monitoring or assessing nutrition [43].

### Gaps Identified in Outcome Evaluation of Mobile Health Technologies

As part of the multidisciplinary assessment, the HTA-CM introduces 9 domains for assessing the outcomes of health technologies, including “health problem and current use of the technology; description and technical characteristics of technology; safety; clinical effectiveness; costs, economic evaluation; ethical analysis; organizational aspects; social aspects; and legal aspects” [11]. The included studies assessed the efficacy of mHealth interventions in health problems and description of the app, clinical effectiveness, partial costs, and economic evaluation. Although the outcome evaluation was multifaceted with respect to the general characteristics and intervention specific of mHealth technologies, a major gap was identified as the lack of evidence on safety, organizational aspects, as well as sociocultural, ethical, and legal aspects in the included studies.

Safety is a major concern for adopting mobile technologies into patient care. In health care, patient safety is paramount. When introducing mHealth solutions into a care setting, the potential impact and threats on patient safety when using a mHealth solution should be scrutinized. Technical safety related to the reliability and validity of evaluating assessment should also be taken into consideration. Privacy, security, collaboration, data sharing, traceability, and transparency are essential for the enhancement of health care services. Technical barriers such as infrastructure, connectivity, bandwidth, resolution, and frame rate associated with data transmission may affect access and accessibility, posing a potential threat of unplanned downtime between patients and practitioners [44].

When searching for studies, we intentionally did not limit the target users of mHealth apps. However, it is worth noting that all the mHealth solutions in the included studies in this review aimed at patients as their primary users, and none of them was designed for renal care providers or caregivers. Furthermore, none of the mHealth solutions was integrated into electronic health record (EHR) systems in their respective care settings. Integrating mHealth interventions into the EHR system might be a major obstacle to the wider adoption of mHealth solutions in renal care.

While utilizing mHealth technologies and implementing this new service, how this new service will fit within the existing organizational framework is an important question and plays a significant role in the evaluation [44]. This may involve changes in business structure and process, business culture, management on workflow and staff, causing extensive organizational changes, as well as the degree of interoperability between information systems and the impact on resource allocation [44]. When a patient performs the dialysis treatment at home, particularly the HD, there is a need for real-time monitoring of vital signs and providing prompt feedback. This may require care providers to work on a different schedule and workloads. How an mHealth solution impacts the care provider’s service and funding model and the level of satisfaction among clinicians should also be assessed?

The significant disease burden owing to loss of time and income often brings many changes to patients with CKD undergoing dialysis in their social and work life with diminished capacities. When planning an mHealth solution, it is important to consider additional responsibility imposed on patients and caregivers by technologies. When introducing mobile technologies into dialysis care, is a patient’s autonomy enhanced or compromised? How will we maintain the equity in access to service among different socioeconomic groups? On a larger scale, the impact on professional accreditation and liability, information governance, and patient privacy in terms of consent and access control will also need to be evaluated [44].

Of note, long-term effects on morbidity, including physical and mental health, were not assessed in any of the included studies. It suggested a need for rigorous RCTs with longer follow-up period to capture relevant data and evaluate these effects. In addition, the evaluation of the cost-effectiveness of mHealth technologies in patients undergoing dialysis is not done comprehensively. For example, the IT resources used and the associated cost for supporting an mHealth app were not discussed in any of the included studies. Regarding the business scenario, the funding models, direct costing, annual expenditures related to the resources, annual revenue associated with the number of patients or services on activity and reimbursement, and the return on investment were not evaluated from the institutional level.

Although the study results revealed positive outcomes in reducing the utilization of health resources and saving costs, it contrasted with the results of a large study conducted by Henderson et al [45] in the United Kingdom, evaluating the cost-effectiveness of telehealth technology in patients with chronic conditions (heart failure, chronic obstructive pulmonary disease, or diabetes), compared with the standard practice. The findings suggested that the quality-adjusted life year gained by patients in the intervention group was similar to that of the control arm; however, the telehealth intervention was associated with higher total costs. Thus, they concluded, “Telehealth does not seem to be a cost-effective addition to standard support and treatment.” [45]. Although their research neither targeted mHealth interventions nor recruited patients undergoing dialysis, as the largest study evaluating telehealth technology from the cost-effectiveness perspective, it did highlight the need for a comprehensive economic evaluation framework when assessing mHealth technologies in health care.

One cohort study indicated a slight decrease in perceived QoL in the intervention group from baseline to the midpoint of the study, whereas scores remained the same as their peers receiving standard care [28]. The 21% dropout rate (5/24 patients in the intervention group) may have contributed to such a result because these dropout patients were representative of a sicker population with potential lower perceived QoL.

### Implications for Future Research

This scoping review revealed the scarcity of evidence or the gap in evaluating the impact of mHealth solutions on care provider’s organizational and legal aspects, as well as patients’ sociocultural and safety aspects for adult patients on chronic dialysis. Importantly, the economic value of mHealth technologies is an integral component in the management of this patient population. Future research could include additional data collection to enable the intent-to-treat analysis of efficacy and cost-benefit [28]. Because limited RCTs have been completed on the topic, this review provides a necessary baseline from which additional trials with larger sample sizes in a variety of patient population analysis is warranted [46].

In addition, this review could inform a co-design process and the development of a comprehensive service model utilizing mHealth technologies for supporting home monitoring and self-management of adult patients undergoing chronic dialysis.

### Limitations

Publication bias was one of the limitations of this study design, for example, non-English publications had been excluded. Consequently, relevant studies initiated in native non-English speaking countries were not captured in this review. Most of the studies included were conducted in developed countries (20/22), which may lead to limited global generalizability. Of the 22 included studies, 15 (68%) originated in North America. Regional discrepancies, clinical and social practices, as well as health care systems and policies, may not be generalizable to other regions.

### Conclusions

The mHealth technologies have been used for adult patients undergoing chronic dialysis, with the main functions being nutrition or dietary self-monitoring, RBM, web-based portal, self-monitoring of in-session dialysis-specific information, and self-monitoring of lifestyle or behavioral change. On the basis of this scoping review, mHealth technologies showed neutral to positive results on patient satisfaction and acceptance, clinical effectiveness, economic assessment, HRQoL, and impact on lifestyle or behavioral change in this cohort.

Despite the potential benefits of utilizing mHealth technologies in patients undergoing dialysis, mHealth solutions have not been widely adopted and integrated into standard renal care. This review highlighted the lack of a comprehensive evaluation that includes a patient’s safety and their sociocultural status, as well as a care provider’s organizational, ethical, and legal aspects when assessing mHealth technologies in care of patients on dialysis. Due to sparse evidence in the literature, the clinical effectiveness and economic effects have not been adequately assessed, especially missing the long-term effects and cost-effectiveness of using mHealth technologies. More rigorous studies in this field continue to be performed, making cost-benefit evaluation a standard process to assist the decision making to an established sustainable business model and organizational support for mHealth implementation in management of patients undergoing dialysis.

## Appendix

Multimedia Appendix 1 Keyword search strategy.

Multimedia Appendix 2 Gray literature search methods.

Multimedia Appendix 3 Characteristics of the included papers.

## Abbreviations

BP: blood pressure
CADTH: Canadian Agency for Drugs and Technologies in Health
CINAHL: The Cumulative Index to Nursing and Allied Health Literature
CKD: chronic kidney disease
EHR: electronic health record
ER: emergency room
HD: hemodialysis
HHD: home hemodialysis
HRQoL: health-related quality of life
HTA: health technology assessment
IWG: interdialytic weight gain
KDQOL: Kidney Disease Quality of Life
mHealth: mobile health
PDA: personal digital assistant
PRISMA: Preferred Reporting Items for Systematic Reviews and Meta-Analysis
PTH: parathyroid hormone
QoL: quality of life
RBM: remote biometric monitoring
RCTs: randomized controlled trials
SF-36: 36-Item Short Form Health Survey
