# Supplementary material for: Intervention and Evaluation of Mobile Health Technologies in Management of Patients Undergoing Chronic Dialysis: Scoping Review
Source: JMIR Mhealth Uhealth. 2020 Apr 3;8(4):e15549. doi: 10.2196/15549 (PMC7165304; doi:10.2196/15549)
Supplement: Multimedia Appendix 1 [file mhealth_v8i4e15549_app1.docx]

## APPENDIX A: KEYWORD SEARCH STRATEGY

| **Concepts** | **(P) Dialysis Population** | **(I) mHealth Intervention** |
| --- | --- | --- |
| Author Keywords | Dialysis  Hemodialysis  Haemodialysis  Hemodialysis  Haemodialyses  HD  HHD  PD | mHealth  “m health”  eHealth  “e Health”  Telehealth  “mobile health”  Mobile app*  Mobile technology  Mobile phone  Smartphone*  Cellular phone*  Cell phone*  Tablet*  Personal digital assistant  PDA  Wearable  Biosensor*  Portable electronic application*  Portable software application*  Text messages  Phone app*  Cellphone app*  Telephone app*  Remote monitoring  Remote patient monitoring  Remote patient management  Remote biometric monitoring  Telenephro*  Tele-nephro*  Telecare  Telecommunication* |
| Subject Headings: MeSH | Kidney failure, chronic[MeSH]  Renal dialysis[MeSH]  Hemodialysis, home[MeSH]  Peritoneal dialysis[MeSH]  Peritoneal Dialysis, Continuous Ambulatory[MeSH]  [Hemodiafiltration](https://www.ncbi.nlm.nih.gov/pubmed/30212979)[MeSH]  [Hemoperfusion](https://www.ncbi.nlm.nih.gov/pubmed/30212979)[MeSH] | Telemedicine[MeSH]  Smartphone[MeSH]  Mobile applications[MeSH] |

**MEDLINE/PubMed Search Statement**

(dialysis[tw] OR hemodialysis[tw] OR haemodialysis[tw] OR hemodialyses[tw] OR haemodialyses[tw] OR HD[tw] OR HHD[tw] OR PD[tw] OR kidney failure, chronic[mesh] OR renal dialysis[mesh] OR hemodialysis, home[mesh] OR peritoneal dialysis[mesh] OR Peritoneal Dialysis, Continuous Ambulatory[MeSH] OR [Hemodiafiltration](https://www.ncbi.nlm.nih.gov/pubmed/30212979)[mesh] OR [Hemoperfusion](https://www.ncbi.nlm.nih.gov/pubmed/30212979)[mesh]) **AND** (mHealth[tw] OR “m health”[tw] OR eHealth[tw] OR “e Health”[tw] OR telehealth[tw] OR “mobile health”[tw] OR mobile app*[tw] OR mobile technology[tw] OR mobile phone*[tw] OR smartphone*[tw] OR cellular phone*[tw] OR cell phone*[tw] OR tablet*[tw] OR personal digital assistant[tw] OR pda[tw] OR wearable[tw] OR biosensor*[tw] OR portable electronic application*[tw] OR portable software application*[tw] OR text messages[tw] OR phone app*[tw] OR cellphone app*[tw] OR telephone app*[tw] OR remote monitoring[tw] OR remote patient monitoring[tw] OR remote patient management[tw] OR remote biometric monitoring[tw] OR telenephro*[tw] OR tele-nephro*[tw] OR telecare[tw] OR telecommunication*[tw] OR telemedicine[MeSH] OR smartphone[MeSH] OR mobile applications[MeSH]) **NOT** (acute kidney injury[tw] OR acute renal failure[tw] OR aki[tw] OR acute kidney failure[tw]) **NOT** wearable artificial kidney[tw] **NOT** (pediatric[tw] OR adolescent[tw] OR child[tw]) **NOT** (parkinson's disease[tw] OR parkinson disease[tw] OR parkinsons disease[tw] OR pd[tw] OR parkinsons[tw] OR parkinsonism[tw])

**Scopus Search Statement**

TITLE-ABS-KEY((dialysis OR hemodialysis OR haemodialysis OR hemodialyses OR haemodialyses OR “chronic kidney failure” OR “renal dialysis” OR “home hemodialysis” OR “home haemodialysis” OR “peritoneal dialysis” OR Hemodiafiltration OR Hemoperfusion) **AND** (mHealth OR “m health” OR eHealth OR “e Health” OR telehealth OR “mobile health” OR “mobile app*” OR “mobile technology” OR “mobile phone*” OR smartphone* OR “cellular phone*” OR “cell phone*” OR tablet* OR “personal digital assistant” OR “PDA” OR wearable OR biosensor* OR “portable electronic application*” OR “portable software application*” OR “text messages” OR “phone app*” OR “cellphone app*” OR “telephone app*” OR “remote monitoring” OR “remote patient monitoring” OR “remote patient management” OR “remote biometric monitoring” OR telenephro* OR tele-nephro* OR telecare OR telecommunication* OR telemedicine) **AND NOT** (“acute kidney injury” OR “acute renal failure” OR “aki” OR “acute kidney failure”) **AND NOT** “wearable artificial kidney” **AND NOT** (pediatric OR adolescent OR child) **AND NOT** (“parkinson’s disease” OR “parkinson disease” OR “parkinsons disease” OR parkinsons OR parkinsonism))

**AND** PUBYEAR > 2007

**CINAHL Search Statement**

dialysis OR hemodialysis OR haemodialysis OR hemodialyses OR haemodialyses OR "HD" OR "HHD" OR "PD" OR “chronic kidney failure” OR “renal dialysis” OR “home hemodialysis” OR “home haemodialysis” OR “peritoneal dialysis” OR Hemodiafiltration OR Hemoperfusion **AND** mHealth OR “m health” OR eHealth OR “e Health” OR telehealth OR “mobile health” OR “mobile app*” OR “mobile technology” OR “mobile phone*” OR smartphone* OR “cellular phone*” OR “cell phone*” OR tablet* OR “personal digital assistant” OR “PDA” OR wearable OR biosensor* OR “portable electronic application*” OR “portable software application*” OR “text messages” OR “phone app*” OR “cellphone app*” OR “telephone app*” OR “remote monitoring” OR “remote patient monitoring” OR “remote patient management” OR “remote biometric monitoring” OR telenephro* OR tele-nephro* OR telecare OR telecommunication* OR telemedicine
