# Supplementary material for: Intervention and Evaluation of Mobile Health Technologies in Management of Patients Undergoing Chronic Dialysis: Scoping Review
Source: JMIR Mhealth Uhealth. 2020 Apr 3;8(4):e15549. doi: 10.2196/15549 (PMC7165304; doi:10.2196/15549)
Supplement: Multimedia Appendix 2 [file mhealth_v8i4e15549_app2.docx]

## APPENDIX B: GREY LITERATURE SEARCH METHODS

**Keywords in search: mobile health dialysis**

| **Organization name** | **No. of items identified for full screening (uploaded to bibliographic software)** |
| --- | --- |
| Canadian Agency for Drugs and Technologies in Health (CADTH) | 14 returned records, none relevant |
| Health Quality Ontario | Zero records returned |
| Google and Google Scholar | Checked the first 40 records, 7 records relevant |
| FDA | Checked the first 40 records, none relevant |
| Ottawa Hospital Research Institute | Zero records returned |
| Pan Canada HTA Collaborative | 26 records returned, 1 relevant |
| International Information Network on New and Emerging Health Technologies | Zero records returned |
