# Supplementary material for: Intervention and Evaluation of Mobile Health Technologies in Management of Patients Undergoing Chronic Dialysis: Scoping Review
Source: JMIR Mhealth Uhealth. 2020 Apr 3;8(4):e15549. doi: 10.2196/15549 (PMC7165304; doi:10.2196/15549)
Supplement: Multimedia Appendix 3 [file mhealth_v8i4e15549_app3.docx]

## APPENDIX C: CHARACTERISTICS OF THE INCLUDED ARTICLES

| **First Author (Year)** | **Study Design** | **Study Location** | **Patient Cohort** | **Study Purpose/**  **Objectives** | **Main Applications** | **Delivery Method** | **Follow-up** | **Data Collection** | **Outcomes Measured** | **Results** |
| --- | --- | --- | --- | --- | --- | --- | --- | --- | --- | --- |
| Connelly et al. (2012) | RCT | USA | HD | Design and evaluation of the dietary intake monitoring app | Nutrition/dietary self-monitoring | PDA | 6 weeks | 1,7 | a | a: Easy to use |
| Dey, Jones, & Spalding (2016) | Prospective Cohort | UK | PD | Explore patient acceptability of technology and evaluate its effect on clinical interventions and QoL | RBM | Computer tablets (PODs) | > 15 months | 1,3,6,7,8 | a,c,d | a: High QUEST scores (NS)  c: No. of admissions avoided, medication change, self-manage at home, phone calls to patients, healthcare professionals visits, referrals/advice  d: NS |
| Hayashi et al. (2017) | Prospective Cohort | Japan | HD (outpatient) | Investigate the feasibility and usability of a novel smartphone-based self-management support system | Self-monitoring of dialysis data | Smartphone & the paired scale | 2 weeks | 1,2,3,6 | a,b,d | a: Positive  b: NS: IWG, serum phosphorus; M: serum potassium  d: SS: social functioning; M: dialysis staff encouragement, vitality; NS: KDQoL |
| Nicdao et al. (2016) | Retrospective Cohort | Australia | HHD | Introduce and evaluate a mobile app 'My Home Hemo' and a web portal for remote monitoring of patients' dialysis parameters to support patients | Online portal | Android (smartphones and tablets) and iOS mobile devices | > 21 weeks | 3,7,8 | a,c | a: Easy to use  c: Decrease: nursing and patient times (travel time, travel distance) |
| Weinhandl & Collins (2018) | Retrospective Cohort | USA | HHD | Compare risks of all-cause and cause-specific HHD attrition between groups; compare the incidence of training graduation | RBM | Tablet | 2 years | 1,3 | b | b: Adjusted hazard ratios, dialysis cessation, technique failure |
| Welch et al. (2013) | Prospective Cohort | USA | HD | Pilot test an electronic self-monitoring intervention based on social cognitive theory | Nutrition/dietary self-monitoring | PDA | 14 weeks | 1,3,5,7 | a,b,e | a: SS: perceived control over time; NS: perceived benefits or self-efficacy, high acceptability score  b: Strong trend: IWG;  e: SS: sodium intake and calories; M: calories and protein |
| Chaudhry, Schaefbauer, Jelen, Siek, & Connelly (2016) | Prospective Cohort | USA | Chronic Dialysis | Explore the usability and feasibility of a dietary intake mobile app that emphasizes portion size estimation | Nutrition/dietary self-monitoring | Mobile phone | 6 weeks | 1,3,5,7 | a | a: Easy to use, easy to understand, satisfactory experience, willing to use again and recommend it to peers |
| Hand, Leon, & Steiber (2014) | RCT (3-arm trial) | USA | HD | Test the feasibility of a 3-arm trial to separate the effects of additional patient care time from the effects of algorithm use | Nutrition/dietary self-monitoring | Tablet computer (handheld tablet) | 6 months | 1,2,3,4 | a,b,e | a: Improved interactions with patients  b: SS: high serum PTH; M: high serum phosphorus; NS: serum phosphorus, PTH  e: Adjusted calcium by group over time |
| Liu et al. (2017) | Mixed method | Australia | HHD | Examine how patients perceive and utilize the functions related to emotion sharing and copresence enhancement | Self-monitoring of dialysis data | Patient-owned mobile device | > 6 months | 1,3 | a,c | a: Patients: ease of use, high reliability and performance, high perceived usefulness, positive feedback regarding the usage; Nurses: satisfaction  c: Savings of nursing and patient times (travel time and travel distance) |
| Harrington et al. (2014) | Prospective Cohort | USA | PD (CAPD) | Examine the effectiveness of using an iPad app to allow real-time and asynchronous monitoring | Self-monitoring of dialysis data | Tablet computer, iPad, owned device | 8 months | 1,3,4,7,8 | a | a: Fair: the Likert evaluation, overall impression of the interface |
| Stark et al. (2011) | RCT | USA | PD & HD | Evaluate a PDA-based dietary intervention designed to moderate dietary sodium intake | Nutrition/dietary self-monitoring | PalmOne Tungsten/E2 PDA | 16 weeks | 1,3,7 | a | a: High adherence |
| Lew, Sikka, Thompson, & Magnus (2018) | Prospective Cohort | USA | PD | Examine whether daily RBM of BP and weight is associated with changes in using hospital/health care services | RBM | RBM | N/A | 1,3,7 | c | c: SS: outpatient visit claim payment, cost for measuring BP/weight, cost for outpatient stays, cost for aged >=55 years and males; M: outpatient visit claim payment (females): NS: outpatient visit claim payment (Black); inpatient costs (females); race; presence of at least one comorbidity associated with cost |
| Nayak, Karopadi, Antony, Sreepada, & Nayak (2012) | Retrospective Cohort | India | PD | Develop a unique PD remote monitoring system | Online portal | Computer or mobile phone using an Android- or iPhone-based application | ~17 months | 1,3,5,7,8 | b | b: SS: 5-year survival; NS: technique failure rates, peritonitis rates, exit-site infections |
| Han et al. (2016) | Prospective Cohort | USA | HD | Assess and quantify PA and sleep levels | Self-monitoring of lifestyle/behavioral change | Fitbit ® Flex TM device | 5 weeks | 1,2,3,4 | a,e | a: Easy to incorporate the activity-tracking device into lifestyle; want to continue wearing it  e: Quantified PA & sleep patterns |
| Kiberd et al. (2018) | Prospective Cohort | Canada | HHD or PD | Determine whether an eHealth portal is effective at improving a patient’s experience with their home dialysis care | Online portal | Computer or mobile device | 1 year | 1,2,3,5,7 | a,c,d | a: Fair to high scores: overall satisfaction, overall satisfaction with the portal, belief in a positive impact on access to a specialist, acceptability, easy to use  c: SS: decrease in phone usage  d: NS: overall continuous quality improvement in nephrologists/nurses/dieticians |
| Magnus, Sikka, Cherian, & Lew (2017) | Prospective Cohort | USA | PD | Evaluate patient satisfaction with and outcomes associated with use of intervention | RBM | HealthPal® device | N/A | 1,3,6 | a | a: SS: satisfied or completely satisfied, completely unsatisfied (decrease), attitudes and perceptions of ESRD and care, confidence/frustration/related perceptions; NS: demographic or clinical characteristics |
| Neumann et al. (2013) | Prospective Cohort | Germany | HD | Examine if body weight telemetry is useful to reduce IWG | RBM | TC 100 Mobil scales, mobile phones | 3 months | 1,2,3,6,7,8 | b | b: SS: IWG for the IDI2, IWG for the unfiltered weekly, mean time duration on dialysis; NS: IWG, ultrafiltration; M: telemetric body weight |
| Sevick et al. (2008) | Case Study | USA | HD | Determine the efficacy of a dietary intervention to reduce dietary sodium intake | Nutrition/dietary self-monitoring | PDA | 16 weeks | 1,2,3 | b,e | b: Decrease: serum phosphorus, IWG, K+ lab value; Unchanged: serum albumin  e: Decrease: sodium, Increase: protein |
| Nayak Karopadi, Antony, Subhramanyam, & Nayak (2013) | Retrospective Cohort | India | PD & HD | Highlight the importance of remote monitoring for a successful PD program | Online portal | mobile phone | 17 months | 1,2,3,7 | c,d | c: SS: erythropoietin cost, hidden costs; NS: basic treatment cost, lab testing cost, complication costs  d: SS: PCS and MCS between PD and HD; NS: depression levels, PCS and MCS between rural and urban |
| Minatodani & Berman (2013) | Prospective Cohort | USA | Chronic dialysis | Determine if the improved outcomes and economic advantages found in the pilot study were reproducible in a larger sample followed for a longer time period | RBM | Home monitoring unit (VitelCare Turtle 500) | 42 months | 1,6,8 | c | c: SS: # of hospitalizations, # of hospital days, hospital and ER costs, hospitalizations, hospital and ER costs; erythropoietin cost & hidden costs; NS: total study days per patient, ER visits, hospital days, ER visits, basic treatment cost, bab testing cost, complication costs; Decrease: # of nurse-initiated telephone contacts |
| Berman et al. (2011) | RCT | USA | HD | Determine whether home-based interventions can improve health outcomes and be economically sustainable to justify their use | RBM | Home monitoring unit (VitelCare Turtle 500) | 1 year | 1,3,7 | b,c,d | b: NS: severity of illness, modified Charlson comorbidity index, risk score  c: SS: frequency of hospitalizations, # of days in the hospital, # of ER visits and associated charges  d: NS: SF-36 scores, perceived QOL (slightly higher) |
| Imtiaz et al. (2017) | Prospective Cohort | Canada | PD | Evaluate OkKidney, a phosphate counting app that matches meal phosphate content with binder dose | Nutrition/dietary self-monitoring | iPod | 30 days | 1,3,6,7 | a,b,e | a: Easy to use, would continue to use, better understanding and better awareness  b: No overall change: serum phosphate, calcium, TRI score; NS: serum phosphate, serum calcium  c: Decrease: calcium carbonate intake; NS: calcium carbonate |

SS: statistically significant with *p* < .05; NS: non-significant with *p* > .05; M: marginal with .05 < *p* < .10

1: Patient characteristics; 2: Clinical characteristics; 3: In-session control/monitoring dialysis-specific information; 4: Symptom control/monitor/management; 5: Lifestyle/behavioral change; 6: QoL assessment; 7: Usage of mHealth applications; 8: Usage of hospital/healthcare services with (or without) costs

a: User Experience/Satisfaction; b: Clinical effects; c: Economic assessment; d: QoL; e: Behavior
